# Responding to a new generation of proprietary study resources in medical education

**DOI:** 10.5195/jmla.2019.619

**Published:** 2019-04-01

**Authors:** Robin O’Hanlon, Gregory Laynor

**Affiliations:** Associate Librarian, User Services, Memorial Sloan Kettering Cancer Center Library, Memorial Sloan Kettering Cancer Center, New York, NY, ohanlonr@mskcc.org; Medical Librarian, Krausz Library of Podiatric Medicine and Ginsburg Health Sciences Library, Temple University School of Podiatric Medicine, Philadelphia, PA, laynor@temple.edu

## Abstract

Traditionally, health sciences libraries have supported patrons who are preparing for medical licensure examinations by collecting and making accessible board exam preparation resources, such as question banks and study guides. However, when online board exam preparation resources are not available for licensing, providing equitable access to all library users can be a challenge. In recent years, a new generation of online study resources has emerged. Sites such as SketchyMedical and Picmonic use visual learning mnemonics, while resources such as Quizlet leverage crowd-sourcing to generate study content. While some of the content from these resources is made freely available, these resources are often limited to paid individual subscribers. This new generation of study resources, thus, presents a conundrum for health sciences librarians. On the one hand, these innovative resources offer new insights into how students learn and study, reflecting pedagogical trends in self-directed learning. On the other hand, the proprietary individual subscription–based model of these resources can widen the achievement gap between students who can afford to pay subscription costs and those who cannot. This commentary provides an overview of some of the most popular medical board examination preparation resources that have emerged in recent years. The authors suggest that health sciences librarians collaborate with medical students and educators to better understand and evaluate these resources.

Any health sciences librarian who is involved in supporting medical education has likely witnessed the cyclical uptick in anxiety among students around the time of licensure examinations. The board exam alphabet soup—United States Medical Licensing Examination (USMLE), National Council Licensure Examination (NCLEX), National Board Dental Examination (NBDE)—has long provoked discussion and debate among health sciences librarians and medical educators on how to best support stressed-out students. Many health sciences libraries license question banks (i.e., BoardVitals, McGraw Hill’s USMLE Easy, ExamMaster) and provide access to exam preparation electronic books (i.e., the *First Aid* series). Many libraries develop board exam LibGuides to highlight study resources. But often, health sciences librarians throw up their hands at high subscription costs or the unwillingness of some vendors to license exam study resources to libraries.

Negotiating with vendors who appear determined to maintain an individual subscription model (and high profit margin) for board exam prep resources can feel like a Sisyphean undertaking. At best, librarians convince outside departments (i.e., medical and nursing education or student services) to collaborate on cost-sharing for site-wide licenses. At worst, libraries are unable to license online resources and are stuck buying individual print copies of exam prep books.

In recent years, a new generation of online board exam prep resources has emerged. When they are available only to individual subscribers, these resources present challenges to health sciences librarians who are committed to making resources accessible to all patrons. Increasingly popular among students, this new generation of study resources embraces pedagogical innovations that transform traditional study approaches such as question banks, study guides, flash cards, and practice tests. These resources are forcing health sciences librarians to consider the question: If students are finding (and individually paying for) new ways to study online for boards, what does that tell us about the limitations of the study resources that we traditionally license and collect? And if we cannot license a resource, should we even bother to learn more about it?

One challenge for health sciences librarians is how to categorize these new resources (Table 1). The authors propose the name “proprietary study resources” for study resources that contain curated content and limit access to individual subscribers. Some of these resources include free content, but only subscribers have full access to all of the features. Related to proprietary study resources (and sometimes overlapping with them) are user-generated and crowd-sourced study resources, such as Quizlet and Course Hero, which aggregate or curate material produced by users themselves.

**Table 1:**
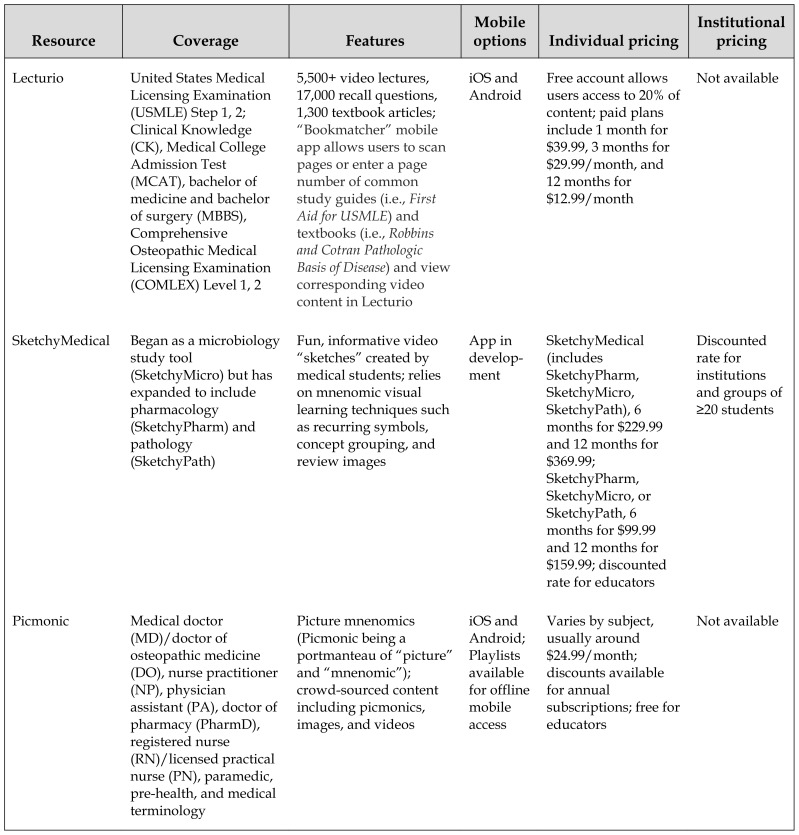

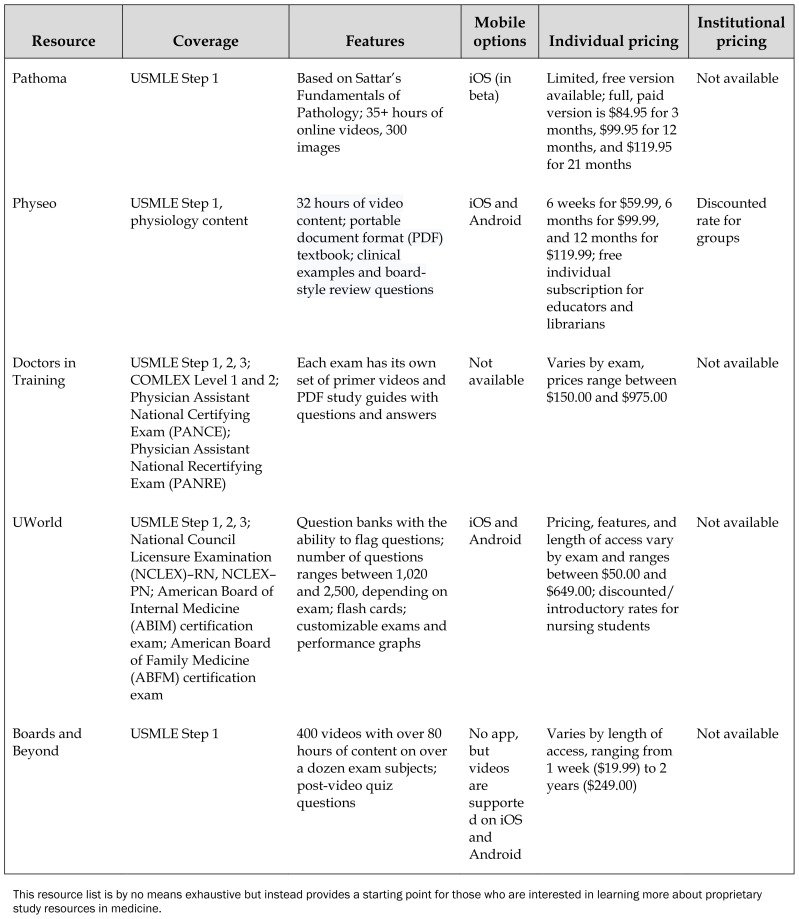
Proprietary study resources in medical education

Resource	Coverage	Features	Mobile options	Individual pricing	Institutional pricing
Lecturio	United States Medical Licensing Examination (USMLE) Step 1, 2; Clinical Knowledge (CK), Medical College Admission Test (MCAT), bachelor of medicine and bachelor of surgery (MBBS), Comprehensive Osteopathic Medical Licensing Examination (COMLEX) Level 1, 2	5,500+ video lectures, 17,000 recall questions, 1,300 textbook articles; “Bookmatcher” mobile app allows users to scan pages or enter a page number of common study guides (i.e., *First Aid for USMLE*) and textbooks (i.e., *Robbins and Cotran Pathologic Basis of Disease*) and view corresponding video content in Lecturio	iOS and Android	Free account allows users access to 20% of content; paid plans include 1 month for $39.99, 3 months for $29.99/month, and 12 months for $12.99/month	Not available
SketchyMedical	Began as a microbiology study tool (SketchyMicro) but has expanded to include pharmacology (SketchyPharm) and pathology (SketchyPath)	Fun, informative video “sketches” created by medical students; relies on mnenomic visual learning techniques such as recurring symbols, concept grouping, and review images	App in develop-ment	SketchyMedical (includes SketchyPharm, SketchyMicro, SketchyPath), 6 months for $229.99 and 12 months for $369.99; SketchyPharm, SketchyMicro, or SketchyPath, 6 months for $99.99 and 12 months for $159.99; discounted rate for educators	Discounted rate for institutions and groups of ≥20 students
Picmonic	Medical doctor (MD)/doctor of osteopathic medicine (DO), nurse practitioner (NP), physician assistant (PA), doctor of pharmacy (PharmD), registered nurse (RN)/licensed practical nurse (PN), paramedic, pre-health, and medical terminology	Picture mnenomics (Picmonic being a portmanteau of “picture” and “mnenomic”); crowd-sourced content including picmonics, images, and videos	iOS and Android; Playlists available for offline mobile access	Varies by subject, usually around $24.99/month; discounts available for annual subscriptions; free for educators	Not available
Pathoma	USMLE Step 1	Based on Sattar’s Fundamentals of Pathology; 35+ hours of online videos, 300 images	iOS (in beta)	Limited, free version available; full, paid version is $84.95 for 3 months, $99.95 for 12 months, and $119.95 for 21 months	Not available
Physeo	USMLE Step 1, physiology content	32 hours of video content; portable document format (PDF) textbook; clinical examples and board-style review questions	iOS and Android	6 weeks for $59.99, 6 months for $99.99, and 12 months for $119.99; free individual subscription for educators and librarians	Discounted rate for groups
Doctors in Training	USMLE Step 1, 2, 3; COMLEX Level 1 and 2; Physician Assistant National Certifying Exam (PANCE); Physician Assistant National Recertifying Exam (PANRE)	Each exam has its own set of primer videos and PDF study guides with questions and answers	Not available	Varies by exam, prices range between $150.00 and $975.00	Not available
UWorld	USMLE Step 1, 2, 3; National Council Licensure Examination (NCLEX)–RN, NCLEX–PN; American Board of Internal Medicine (ABIM) certification exam; American Board of Family Medicine (ABFM) certification exam	Question banks with the ability to flag questions; number of questions ranges between 1,020 and 2,500, depending on exam; flash cards; customizable exams and performance graphs	iOS and Android	Pricing, features, and length of access vary by exam and ranges between $50.00 and $649.00; discounted/introductory rates for nursing students	Not available
Boards and Beyond	USMLE Step 1	400 videos with over 80 hours of content on over a dozen exam subjects; post-video quiz questions	No app, but videos are supported on iOS and Android	Varies by length of access, ranging from 1 week ($19.99) to 2 years ($249.00)	Not available

This resource list is by no means exhaustive but instead provides a starting point for those who are interested in learning more about proprietary study resources in medicine.

While proprietary study resources might seem to be the opposite of open education resources that aim to make content available for free to all, both are part of broader changes in the creation and delivery of medical education content. Proprietary study resources, like crowd-sourced study resources and open education resources, are part of a shift to more self-directed student learning in medical education. In the era of flipped classrooms, students have taken on a more active role in finding, utilizing, and even creating study materials. Proprietary study resources capitalize on this trend.

In an April 2017 letter to the editor published in *Academic Medicine*, a third-year medical student at the University of Cincinnati College of Medicine argued that “in this fully digital age, the best teachers of any subject are available to any medical student, anywhere in the world, at any time through Web resources such as Pathoma, SketchyMedical, and others” [1]. Based on the availability of these new resources, the student even went so far as to question “the value of a traditional preclinical basic science curriculum that relies on locally created and delivered content” and suggested that medical educators “redirect their energies towards curricular elements not deliverable at a distance,” instead of reproducing standardized content. This letter reflects some of the overall shifts in medical education, such as the “flipped classroom,” and points to further developments toward “curriculum customization and innovation—graduating learners with deeper experience in research, quality improvement, patient safety, leadership development, and beyond.” As students become more used to teaching themselves and each other, it is not surprising that they are drawn to the new generation of proprietary study resources when they prepare for boards.

But as students turn to these resources, they are challenging some of the assumptions of the library profession. Existing ways of thinking about collections in health sciences libraries often do not fit the new generation of study resources. That students are willing to pay for proprietary study resources is a message to us that the kinds of study resources that we are used to collecting might not match how students are now learning. Medical students have become more accustomed to active learning strategies and multisensory modes of learning [2]. Traditional study resources have not necessarily kept up with the new study habits and learning styles that have emerged in the more visual and interactive digital environment in which today’s students have grown up. As librarians, we ask ourselves: Are we collecting what students are actually using and how they actually learn? If the answer was yes, students would not need to look elsewhere. But they are looking elsewhere, often paying for study resources rather than using resources that are available to them for free in our libraries.

What we thought we knew about board exam study resources might be wrong, and that is okay. Lest the authors draw the ire of librarians who see value in traditional collection practices for providing board exam support, let us be clear that we are not suggesting that you do away with providing access to the resources that you currently make available to your patrons simply because new resources have emerged. We are not advocating you cancel your BoardVitals subscription tomorrow or strip all the *First Aid* e-book records from your catalog. What we are suggesting is a critical evaluation of how we, as health sciences librarians, have traditionally thought about and interacted with these resources. If your patrons are using the study resources that you currently provide and are happy with them, more power to you. However, if they are not or heavily use only one or two resources, it may be time to reevaluate some traditional trains of thought about study resources. Here we outline three trains of thought that health sciences librarians tend to have when faced with students turning to proprietary study resources.

**“We already collect the right stuff, but our patrons do not realize this.”**

Librarians assume that we are providing the best question banks and study guides available. Yet, when usage lags, we tell ourselves that it must be that our users simply cannot find our resources. Library websites are, after all, notoriously cumbersome and overloaded with information. The problem, we assure ourselves, is a lack of awareness stemming from poor marketing on our part and poor usability on the part of our online presence. We resign ourselves to doubling down on promotional efforts, showcasing our study resources on our websites, in the classroom, at committee meetings, and during orientations. Yet, some resources never gain traction.

While there is always room for improvement with our discovery systems, is it really the case that our libraries have all the “right stuff,” but our students cannot find it? The authors posit that the answer is no. Health sciences students are, in many ways, sophisticated users of online content. They take their exams online, view classes online via lecture capture, and even develop their own online study tools. Certainly, they may not be expert Medical Subject Headings (MeSH) searchers, but one quality that students preparing for medical licensure seem to share is a dogged determination to find the right combination of study resources to prepare for their boards. This is evidenced not only by the hundreds of message boards and online communities devoted entirely to this topic, but also by our own experiences in our libraries. How many times have we heard students say, “I’ll focus on my other coursework (this list could be expanded to include a litany of other items including research, personal relationships, overall health and hygiene, etc.) *after* I finish Boards.”

The pressure of passing boards—and fear of failure—looms so large throughout medical education that it, for better or worse, becomes all-encompassing for test-takers and ferreting out the best study resources available becomes a full-time occupation. At one of the author’s institutions, students organized a Boards Committee to share tips about study resources and developed a survey of third-year students to gather information on what study resources worked best when preparing for boards. One student even created his own set of flash cards and began selling them on Amazon. Students being content creators rather than passive consumers of study resources is nothing new. Whether working in groups or individually, students have always cobbled together and passed around their own versions of study guides and flash cards. Resources like SketchyMedical are in some ways the result of the commercialization of these student-made study resources.

**“If vendors won’t license resources to us, we should ignore these resources. If we ignore them, they won’t exist. If we don’t provide access to them, students won’t request them.”**

If a study resource is only available from vendors who only will sell to individual student subscribers in order to maximize profit, health sciences librarians do not have to simply ignore the existence of the resource. To begin addressing barriers to access that heighten and reinforce inequities and readiness, we must at least acknowledge the existence of proprietary study resources.

The nagging reality remains that simply ignoring something does not make it go away. Let us learn from our history: when Wikipedia emerged over fifteen years ago, many librarians followed the “pretend it doesn’t exist” train of thought and others railed against its use. Over time, our profession’s relationship with Wikipedia has evolved: some remain skeptical, some ambivalent, some have embraced its use and have leveraged Wikipedia as a tool for learning or for amplifying the stories of underrepresented groups. Wikipedia has not gone away. Nor will proprietary study resources for medical licensure, especially because these resources, unlike Wikipedia, can generate significant profit for vendors. We will be in a better position to confront inequity and inaccessibility in proprietary study resources if we acknowledge their existence and begin a conversation about how to critically engage with these resources.

**“We shouldn’t acquire or promote resources that have not been fully vetted by medical educators. These resources might interfere with the curricular and pedagogical goals of our institutions.”**

Students are already looking outside the recommendations of faculty members and beyond library collections to find study resources. Students are finding study resources that work for their learning styles and study habits. As one of the frames in the Association of College & Research Libraries’ *Framework for Information Literacy for Higher Education* states: “Authority is constructed and contextual.” Information literacy now requires that students “understand the increasingly social nature of the information ecosystem where authorities actively connect with one another and sources develop over time” [3]. The “authorities” involved in study resources are not just medical educators and librarians, they are also the students who are “vetting” resources based on their usefulness. Instead of automatically dismissing proprietary study resources as unauthorized, health sciences librarians can begin collaborating with students and faculty to develop frameworks for evaluating the new generation of study resources.

As frustrating or confusing as proprietary study resources might be to some of our traditional paradigms, our profession has more to offer when engaging with these resources than by ignoring them. With their innovative uses of graphics and video, these resources can also help us understand how students now learn and why these new resources appeal to their study habits.

Health sciences librarians are well positioned to embrace the challenge of proprietary study resources. Point-of-care tools are an example of how information delivery has changed to meet the needs of users, and the same can happen with study resources. The authors suggest five steps that health sciences librarians can take to critically engage proprietary study resources:

1. Identify the resources that students are using (i.e., who makes them, where does the content come from).
2. Investigate why these resources appeal to students.
3. Collaborate with medical educators to evaluate these resources.
4. Reconsider what kinds of study resources are acquired and supported.
5. Support open education resources as equitable alternatives to proprietary study resources.

Proprietary study resources present challenges that health sciences librarians might not be able to address on our own, without involving additional decision makers in our institutions. One potential opportunity is simply bringing awareness of these tools to faculty and administrators at our institutions. Do they know their learners are using these resources? If so, are there steps that can be taken institutionally to improve equity of access? Can they lobby companies that provide these resources to negotiate with libraries?

Another question we should ask ourselves is, even if we had unlimited budgets, would we want, simply on principal, to license resources like Lecturio, which demands that institutions shell out $300 per student for their content? And if we are not providing access to these resources, should we still refer students to them? Again, the traditional library train of thought would be “no,” but as discussed previously, students will probably discover these resources on their own. One possibility is to include these resources on a study resource LibGuide but also to include a disclaimer. For example, Charlotte Edwards Maguire Medical Library at Florida State University has a “Textbooks & More: Support Materials” LibGuide that has a section on “Peer-Recommended Resources,” which includes resources like Picmonic, Anki (a digital flashcard program), and SketchyMedical, with dollar signs next to each resource to indicate their individual subscription costs [4].

We can also encourage medical educators to help students evaluate these resources. Just like traditional information sources, students must work to critically evaluate their study resources. We can work with students to consider a variety of study resources and emphasize that there probably is not one catch-all resource to meet all their study needs. Learners must be aware of a study resources’ currentness, provenance, and the intentions of its creators (i.e., Is it to make a profit? If so, what are the implications?).

Finally, we should investigate how these resources can be more “open” and how to make it feasible for educators to develop their own open educational study resources for medical licensure. As advocates of open pedagogy and open information, librarians have been at the forefront of open access, open educational resources, and open courseware movements. The possibility of open board exam study resources for medical licensure seems ripe for further exploration.

Proprietary study resources are not going away. The authors advocate for more awareness of these resources and more attention to how and why students use them. Health sciences librarians can help keep channels of communication open between students and faculty and identify potential collaborations. Paying attention to the new generation of study resources raises larger questions such as: How are students studying? What resources are students actually using to study? What are the implications for medical education and for libraries? In addition to paying for proprietary resources, what kinds of user-generated and crowd-sourced content are students using and producing? There are no easy answers to these questions, but we can help begin the conversation.
